# Caveolin-1-deficient fibroblasts promote migration, invasion, and stemness via activating the TGF-β/Smad signaling pathway in breast cancer cells: Caveolin-1-deficient fibroblasts promote breast cancer cell progression

**DOI:** 10.3724/abbs.2022150

**Published:** 2022-11-21

**Authors:** Qingyun Huang, Longyuan Wu, Yi Wang, Xinyu Kong, Xinhua Xiao, Qiyuan Huang, Miao Li, Yujia Zhai, Fuxiu Shi, Ruichen Zhao, Junpei Zhong, Lixia Xiong

**Affiliations:** 1 Department of Pathophysiology Medical College Nanchang University Nanchang 330006 China; 2 The First Affiliated Hospital Nanchang University Nanchang 330006 China; 3 Key Laboratory of Functional and Clinical Translational Medicine Fujian Province University Xiamen 361023 China

**Keywords:** caveolin 1, cancer-associated fibroblasts, breast neoplasms, epithelial-mesenchymal transition, transforming growth factor-β

## Abstract

Cancer-associated fibroblasts (CAFs) represent one of the main components in the tumor stroma and play a key role in breast cancer progression. Transforming growth factor-β (TGF-β) has been established to mediate breast cancer metastasis by regulating the epithelial-mesenchymal transition (EMT) and stemness of cancer cells. Caveolin-1 (CAV-1) is a scaffold protein of caveolae that is related to the proliferation and metabolism of cancer cells. It is now well demonstrated that CAV-1 deficiency in the tumor stroma is positively correlated with distant metastasis, but the mechanism remains unclear. Here, we explore whether CAV-1-deficient fibroblasts play an essential role in the EMT and stemness of breast cancer cells (BCCs) through TGF-β signaling. We establish a specific small interfering RNA (siRNA) to inhibit CAV-1 expression in fibroblasts and coculture them with BCCs to investigate the effect of CAV‑1-deficient fibroblasts and the tumor microenvironment on breast cancer progression. This study refreshingly points out that CAV-1 deficiency in fibroblasts enhances TGF-β1 secretion and then activates the TGF-β1/Smad signaling pathway of BCCs, thus promoting the metastasis and stemness of BCCs. Collectively, our findings indicate an unexpected role of CAV-1 deficiency in fibroblasts and the tumor microenvironment as a permissive factor, which is regulated by the TGF-β1 signaling pathway in BCCs.

## Introduction

According to global cancer statistics in 2021, breast cancer has the highest incidence among women, constituting 30% of all newly diagnosed cancers [1]. Although great progress has been made in the early detection and treatment of breast cancer, its mortality remains the second-highest among female cancers, mostly due to the distant metastasis of breast cancer [2].

Cancer-associated fibroblasts (CAFs) were discovered to be activated fibroblasts, and they represent one of the major components in the tumor microenvironment (TME) [3]. Some researchers have shown that CAFs play an essential role in cancer development and distant metastasis. In breast cancer, CAFs promote its occurrence, metastasis, recurrence, and drug resistance, and they can also be used for clinical diagnosis [4]. Recent studies have suggested that in the TME, CAFs can secrete growth factors, cytokines, and chemokines, thus promoting tumor growth, metastasis, angiogenesis, and establishing an immunosuppressive environment via paracrine mechanisms [ 5‒ 8] . Moreover, fibroblasts (including human embryonic skin fibroblasts) were also used to simulate the TME to explore the interaction axis between CAFs and other components in the TME [ 9‒ 11] .

CAV-1 is the most important functional protein of the caveolin family and the scaffold protein of caveolae, which can bind some signal proteins and control crucial cell functions in organisms, such as endocytosis, signal transduction, cholesterol balance, cell cycle, proliferation, apoptosis, and invasion and metastasis of cancer cells [ 12, 13] . The role of CAV-1 in cancer progression might be linked to the different physiological functions of breast cancer in different stages [14]. Meanwhile, the CAV-1 expression level is also closely related to the progression, distant metastasis, therapeutic resistance, and different outcomes of the patients [ 15‒ 19] , especially the subtype of epidermal growth factor receptor (EGFR)-positive breast cancer patients [20]. Among those, attention has been given to the essential role of CAV-1 in the occurrence and progression of breast cancer and as a potential therapeutic target [21].

According to the study by Shi *et al*. [22], the downregulation of CAV-1 in cocultured fibroblasts can promote proliferation and inhibit the apoptosis of breast cancer cells (BCCs). In addition, the clinical and experimental data of Simpkins *et al*. [23] showed that CAV-1 deficiency in CAFs can enhance the invasiveness of BCCs and is related to the prognosis of breast cancer patients. Moreover, CAV-1 deficiency in prostate cancer stromal cells can accelerate tumor growth and reduce relapse-free survival of patients by upregulating Akt phosphorylation, resulting in oncogenic gene expression, including transforming growth factor-β1 (TGF-β1) [24]. Thus, the CAV-1 expression level in breast cancer tissues might be a predictive biomarker for breast cancer.

Epithelial-mesenchymal transition (EMT) refers to a variety of biological changes that equip epithelial cells with the phenotypes of mesenchymal cells, including reduced intercellular adhesion and enhanced migration and invasion. The specific manifestation involves biomarker transformation from E-cadherin to N-cadherin, which is considered a potential mechanism of tumor metastasis. Meanwhile, cancer stem cells (CSCs) are a class of cancer cells that have the abilities of self-renewal and multidirectional differentiation. This represents the first time that the existence of breast cancer stem cells (BCSCs) was confirmed in breast cancer, with a phenotype of CD44 ^+^ CD24 ^−/low^ [25]. B-cell-specific Moloney murine leukemia virus integration site 1 (BMI1) and SRY (sex-determining region Y)-box 2 (SOX2) are also important biomarkers of CSCs.

Recently, several studies have revealed the inextricable link between EMT and CSCs. EMT can promote the formation and differentiation of CSCs [26]. Furthermore, the adhesion of CSCs decreases, while their motor ability increases, playing an important role in tumor invasion and metastasis [27]. Some studies have demonstrated the connection of TGF-β with both EMT and CSCs. TGF-β plays an important regulatory role in the process of EMT. It can inhibit cancer development in the early stage of tumorigenesis but promote it in the late stage [28]. In addition, it can promote CSCs by interacting with Wnt, human epidermal growth factor receptor 2 (HER2), and focal adhesion kinase (FAK) [29].

Due to the unclear link between CAV-1 deficiency and the effect of TGF-β on the tumor microenvironment and poor patient prognosis, our study focused on the following aspects: (1) to observe the relationship between CAV-1 expression in the tumor stroma and breast cancer progression through analysing database and clinical tissue specimens; (2) to establish a fibroblast model with CAV-1 downregulation; (3) to simulate the breast cancer microenvironment by establishing a coculture model of fibroblasts and BCCs; (4) to explore the correlation between CAV-1-downregulated fibroblasts and the migration and invasion of BCCs, investigate the occurrence of cancer cell EMT, and identify whether they could obtain characteristics of stem cells; and (5) to verify the signaling pathway involved when CAV-1 downregulation in fibroblasts affect the migration and invasion of BCCs.

## Materials and Methods

### Reagents

Anti-caveolin-1, anti-TGF-β1, anti-TGF-β receptor 2 (TGF-βR2), anti-Smad2, anti-p-Smad2, anti-CD44, anti-BMI1, anti-SOX2, anti-E-cadherin, anti-N-cadherin, and anti-vimentin antibodies were purchased from Affinity Biosciences (Cincinnati, USA). Anti-β-actin antibody, HRP-labelled anti-rabbit IgG of goat, and HRP-labelled anti-rat IgG of goat were purchased from Zhongshan Jinqiao Company (Beijing, China). An enzyme-linked immunosorbent assay (ELISA) kit for TGF-β1 was purchased from Elabscience (Wuhan, China). The TGF-β1 inhibitor SB431542 (5 mg) was obtained from Sigma (St Louis, USA). Dulbecco’s modified Eagle’s medium (DMEM) containing 4.5 g/L glucose was obtained from HyClone (Logan, USA). Bovine serum albumin (BSA), dimethylsulfoxide (DMSO), and penicillin/streptomycin were purchased from Solarbio (Beijing, China). Fetal bovine serum (FBS) was purchased from Biological Industries (Cromwell, USA).

### Cell lines and co-culture assays

Human embryonic skin fibroblasts (ESF) were purchased from the Institute of Basic Medicine, Chinese Academy of Medical Sciences (Beijing, China); human breast cancer cell lines (MAD-MB231 and MCF-7) were purchased from Shanghai Cell Bank, Chinese Academy of Sciences (Shanghai, China).

MCF-7 or MAD-MB231 cells (5×10 ^5^) were seeded on a 6-well plate in 2 mL complete media (DMEM, Hyclone, USA) supplemented with 10% FBS (ExCell Bio, Shanghai, China) and 1% penicillin/streptomycin, and fibroblasts (2×10 ^5^) were seeded on the 0.4 mm polyester membrane of a 6-mm Transwell insert (Corning, Lowell, USA) in 2 mL complete media and placed in a separate culture plate. After 24 h, Transwell insert was subsequently transferred to the culture plate with MCF-7 or MDA-MB-231 cells, and the cells were cultured in 2 mL of complete medium at 37°C in a humidified incubator containing 5% CO _2_. The up and down position of human breast cancer cell lines and ESF cells can be switched according to subsequent experiments.

### Experimental groups

Surgical resection specimens were collected from the Pathology Department of the First Affiliated Hospital of Nanchang University from February 2019 to February 2020. Specimens that were pathologically diagnosed including 10 cases with lymph node metastasis and 10 cases without lymph node metastasis were selected. Breast cancer specimens were grouped according to the presence or absence of lymph node metastasis. Then, the expressions of CAV-1, EMT, and cell stemness markers were measured in each group.

In the *in vitro* experiments, MDA-MB-231 cells were grouped into the following four groups: MDA-MB-231 group (MDA-MB-231 cells were cultured separately), MDA-MB-231/ESF group (MDA-MB-231 cells and ESF cells were co-cultured), MDA-MB-231/ESF+NC (negative control) group (MDA-MB-231 cells and ESF cells transfected with negative control were co-cultured), MDA-MB-231/ESF+siRNA308 group (MDA-MB-231 cells and ESF cells transfected with caveolin-1-homo308 were co-cultured). MCF-7 cells were grouped into the following four groups: MCF-7 group (MCF-7 cells were cultured separately), MCF-7/ESF group (MCF-7 cells and ESF cells were co-cultured), MCF-7/ESF+NC group (MCF-7 cells and ESF cells transfected with negative control were co-cultured), MCF-7/ESF+siRNA308 group (MCF-7 cells and ESF cells transfected with caveolin-1-homo308 were co-cultured).

To investigate the secretion of TGF-β1 from fibroblasts, the following experimental groups, including the ESF group (ESF cells were cultured separately), ESF+NC group (ESF cells were transfected with negative control), and ESF+siRNA308 group (ESF cells were transfected with caveolin-1-homo308) were set up.

All cell groups used in this study are summarized as follows: MDA-MB-231 group, MDA-MB-231/ESF group, MDA-MB-231/ESF+NC group, MDA-MB-231/ESF+siRNA308 group, ESF group, ESF+NC group, ESF+siRNA308 group, MCF-7 group, MCF-7/ESF group, MCF-7/ESF+NC group, MCF-7/ESF+siRNA308 group.

### Cell transfection

ESF cells were seeded on a 6-well plate and cultured for 24 h with 2 mL of complete medium per well in advance. The cells were used for cell transfection when they covered 50% to 70% of the wells. ESF cells were transfected with the following siRNAs: caveolin-1-homo526, caveolin-1-homo439, caveolin-1-homo308, and negative control (Suzhou Genepharma Co., Ltd., Shanghai, China). The sequences are shown in Table 1. Lipofectamine 3000 (Invitrogen, Carlsbad, USA) was used for transfection according to the manufacturer’s instructions. After 24 to 48 h, ESFs were used for further experiments.

**Table:** **Table 1** The siRNA nucleotide sequences of NC and Caveolin-1

siRNA	Sequence (5′→3′)
Negative control	UUCUCCGAACGUGUCACGUTT
	ACGUGACACGUUCGGAGAATT
Caveolin1-homo-526	GCAUUUGGAAGGCCAGCUUTT
	AAGCUGGCCUUCCAAAUGCTT
Caveolin1-homo-439	GCGACCCUAAACACCUCAATT
	UUGAGGUGUUUAGGGUCGCTT
Caveolin1-homo-308	GGGACAUCUCUACACCGUUTT
	AACGGUGUAGAGAUGUCCCTT

### Western blot analysis

Cell proteins were extracted using RIPA protein extraction reagent (Beyotime, Shanghai, China) according to the manufacturer’s instructions. The protein concentration was detected using a BCA Protein Assay Kit (Beyotime). The protein samples were balanced to achieve the same concentration. The protein extract was separated by sodium dodecyl sulfonate polyacrylamide gel electrophoresis (SDS–PAGE), and then transferred to polyvinylidene difluoride (PVDF) membranes (Merck Millipore, Billerica, USA). The membranes were blocked and incubated with a specific antibody at 4°C overnight, followed by incubation with the corresponding secondary antibody for 2 h at room temperature. Finally, the immunoreactive protein band was detected using the EasySee Western Blot kit (TransGen Biotech, Beijing, China).

### RT-qPCR

RNA was extracted from cells using a Total RNA Kit II (Omega Bio-Tek, Norcross, USA). EasyScript® One-Step gDNA Removal and cDNA Synthesis SuperMix (TransGen) was used to synthesize cDNA with RNA as a template. Reverse transcription was performed on a ProFlex™ PCR instrument (Applied Biosystems, Foster City, USA) under the following conditions: 42°C for 15 min, 85°C for 5 s. The qPCR system was prepared according to the instructions of the TransStart Tip Green qPCR SuperMix (+Dye II) kit (TransGen). qPCR was performed on an ABI 7500 Real-Time PCR system (Applied Biosystems) under the following conditions: 94°C for 30 s and 40 cycles of 94°C for 5 s and 62°C for 30 s. All target gene expression levels were normalized to that of D-glyceraldehyde-3-phosphate dehydrogenase ( *GAPDH*). All primer pairs were synthesized by BGI (Shenzhen, China), and the sequences are shown in Table 2.

**Table:** **Table 2** Sequence of primers used in quantitative RT-PCR

Gene	Primer sequence (5′→3′)
*Caveolin-1*	Forward CAGTGCATCAGCCGTGTCTA
Reverse TCTGCAAGTTGATGCGGACA
*GAPDH*	Forward TCATCATCTCTGCCCCCTCT
Reverse AGTGATGGCATGGACTGTGG
*Vimentin*	Forward GGACCAGCTAACCAACGACA
Reverse AAGGTCAAGACGTGCCAGAG
*E-cadherin*	Forward GCTGGACCGAGAGAGTTTCC
Reverse CAAAATCCAAGCCCGTGGTG
*SOX2*	Forward GCCCTGCAGTACAACTCCAT
Reverse GACTTGACCACCGAACCCAT
*CD44*	Forward GACAGAGCCACAAGCTTCCA
Reverse CCCTGTTGTCGAATGGGAGT
*BMI1*	Forward GACAGAGCCACAAGCTTCCA
Reverse CCAATACGCCGCAACTCTTG
*Twist1*	Forward CTCGGACAAGCTGAGCAAGA
Reverse GCTCTGGAGGACCTGGTAGA

### Cell scratch test and transwell assays

The transfected ESFs were seeded on a 24-well Transwell plate (8 μm pore size; Corning), and MCF-7 or MDA-MB-231 cells were seeded on a 24-well Transwell plate. For invasion detection, Matrigel was laid on the bottom of the upper chamber 2 h in advance using serum-free medium to simulate the cell-matrix environment. For migration detection, Matrigel is not needed. After the MCF-7 and MDA-MB-231 cells were cultured for 6 h, the cells were placed in a 24-well plate for co-cultivation. After 24‒48 h, the upper chamber was removed, the cells at the bottom of the chamber were washed, fixed, stained, and photographed, and the differences between the groups were compared.

MCF-7 or MDA-MB-231 cells (5×10 ^5^) were seeded in a 6-well Transwell plate and co-cultured with the transfected ESFs for 24‒48 h. The upper chamber was removed, and the cells were washed with PBS. A pipette tip was used to scribe a line perpendicular to the bottom, and cell debris was removed. The plate was incubated in a 37°C 5% CO _2_ incubator, and pictures were taken at 0, 24, and 48 h.

### ELISA

ESF cells (2×10 ^5^) were seeded into a 6-well plate. After ESF cells grew to approximately 50%–70% confluence, cell transfection experiments were performed, and the medium was changed 6 h later. ESF cells are cocultured with MDA-MB-231 or MCF-7-cell lines. After 48 h, the upper chamber of the coculture system was removed. The cell supernatant of each group was collected and centrifuged at 1000 *g* for 20 min at 4°C. After centrifugation, the supernatant was removed for the next step. According to the instructions, the samples of each group were tested using the ELISA kit for TGF-β1 (Elabscience), and the optical density (OD) values were measured at a wavelength of 450 nm with a multifunctional plate reader (Thermo Forma Scientific, Waltham, USA).

### Immunofluorescence staining

Sterile polylysine cell slides were placed in a 6-well plate, and ESF cells were seeded in the plate. The cells were grown to cover 50% of the well, and transient transfection was performed. MDA-MB-231 or MCF-7 cells were seeded into the upper chamber of the coculture chamber. After 48 h of co-culture, the upper coculture chamber and ESF cell culture media were removed. After three times wash with PBS, the fixative solution (methanol:acetone=1:1) was added to the cells and fixed at room temperature for 30 min. TBST (Tris buffered saline containing Tween 20) containing 5% BSA was added and bolcked at 37°C for 2 h. Then, primary antibody was diluted at a ratio of 1:100 and added to the 6-well plate. The cells were incubated with the primary antibody at 4°C overnight. After three times (10 min each time) wash with PBS, the fluorescent secondary antibody was added at a dilution of 1:100 and incubated at 37°C for 2 h in the dark. The cell slide was removed, rinsed with PBS, and stained with DAPI (Invitrogen) to visualize the cell nuclei. Images were taken with an Olympus Th4-200 microscopy system (Olympus, Tokyo, Japan).

### Immunohistochemical (IHC) staining

Breast cancer and para-cancerous specimens were collected from the Pathology Department of the First Affiliated Hospital of Nanchang University. The expression of CAV-1 in breast cancer specimens and adjacent specimens was detected by immunohistochemical staining. All tissues were fixed in 10% neutral buffered formalin for 24–48 h at room temperature and then embedded in paraffin. The tissues were deparaffinized and hydrated by standard procedures. The endogenous peroxidase activity was eliminated with 3% H _2_O _2_. The sections were washed three times (5 min each time) with PBS and soaked in 100°C citrate buffer (pH 6.0) for 40 min to complete antigen retrieval. The specimens were washed three times (5 min each time) with PBS and incubated with primary antibody (diluted at 1:100) in a buffer containing 5% BSA overnight at 4°C. The specimens were then incubated with a biotin-labelled secondary antibody for 30 min at room temperature. Horseradish peroxidase-labelled streptavidin working solution was added to the specimen and incubated at room temperature for 20 min. The samples were washed 3 times (5 min each time) with PBS, and then stained with DAB (diaminobenzidine; Sigma). Next, the specimens were counterstained with hematoxylin, dehydrated, sealed in permanent medium Entellan (Merck, Darmstadt, Germany), and images were captured by a microscope.

### Colony formation assay

Cells in the logarithmic growth phase were trypsinized, resuspend in the complete medium, and inoculated in a 6-well plate at 700–1,000 cells/well. Cells were cultured for 14 days or until the number of cells in a single clone is greater than 50, with medium changed every 3 days. Then cells were washed once with PBS, and 1 mL of 4% paraformaldehyde was added to each well for 30 min fixation. After the cells were washed twice with PBS, 1 mL of crystal violet staining solution was added to each well and incubated for 20 min, followed by several times wash with PBS. Finally, images were captured with a digital camera and the number of colonies in each well was counted.

### Statistical analysis

All data in this study were presented as the mean ± standard deviation (SD) from the results of three or more independent experiments. GraphPad Primer 6 software was used to draw the charts, and SPSS 17.0 software was used to analyze the experimental data. The results were analyzed using the *t* test and chi-square test. Comparisons among multiple groups were analysed by one-way analysis of variance (ANOVA). The difference was considered statistically significant when the *P* value was less than 0.05.

## Results

### CAV-1 deficiency in breast cancer tissues promotes lymph node metastasis

**Figure 1:**
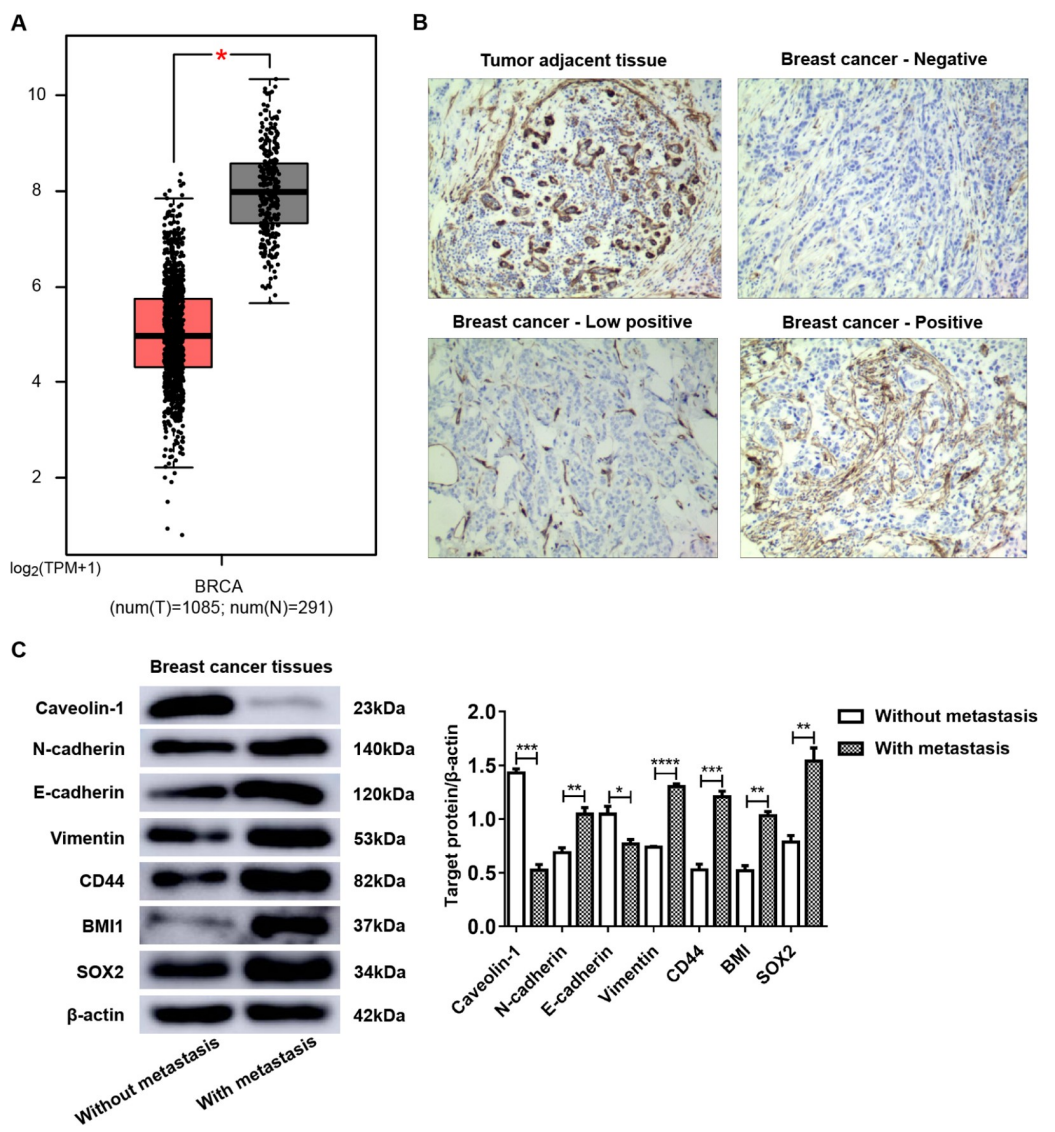
Figure 1 CAV-1 deficiency in breast cancer tissues promotes lymph node metastasis (A) According to the GEPIA data, the CAV-1 expression level in breast cancer tissues is significantly lower than that in normal tissues ( P<0.05). (B) Normal expression, negative expression (–), low positive expression (+), and positive expression (++) of CAV-1 in adjacent and breast cancer stromal tissues. Magnification fold: 100×. (C) The expression levels of EMT and stemness-related proteins, including CAV-1, N-cadherin, E-cadherin, Vimentin, CD44, BMI1, and SOX2, in the breast tumor tissue of 20 cases were elevated by western blot analyses. All these data showed a significant difference between adjacent and breast cancer tissues. * P<0.05, ** P<0.01, and *** P<0.001, compared with the corresponding controls.

According to the data from GEPIA [30], the CAV-1 expression level in breast cancer tissues was significantly lower than that in normal tissues ( *P*<0.05, Figure 1A). To explore whether decreased CAV-1 expression in breast cancer tissue is related to lymph node metastasis, we collected 20 invasive breast cancer tissue specimens from the First Affiliated Hospital of Nanchang University from February 2019 to February 2020. All of these tissues were confirmed by pathological diagnosis, including 10 cases with lymph node metastasis and 10 without. The expression of CAV-1 in tumor tissues was detected by immunohistochemical staining. It was found that the reduction in CAV-1 expression was mainly in the stroma of breast cancer, not in breast cancer parenchyma ( Figure 1B). At 100× magnification, CAV-1-stained stromal fibroblasts were counted and graded, and the results are shown in Table 3. Collectively, the positive rate of CAV-1 expression in breast cancer tissues was 20%, which indicated lymph node metastasis. Nevertheless, in breast cancer without lymph node metastasis, the positive rate reached 90%.

**Table:** **Table 3** Immunohistochemical staining of the expression of CAV-1 in tumor stromal tissues with and without lymph node metastasis

CAV-1 expression in tumor stromal	With lymph node metastasis (+)	Without lymph node metastasis (−)	Summary
Negative (−)	5	0	5
Positive (+)	5	10	15
Summary	10	10	20

To investigate whether the difference in breast cancer lymph node metastasis is correlated with EMT and cell stemness, western blot analysis was performed to measure the expression levels of CAV-1 and EMT and stemness-related proteins in 20 cases of collected breast cancer tissues ( Supplementary Figure S1). As shown in Figure 1C, CAV-1 expression in breast cancer tissues with lymph node metastasis was lower than that in breast tissues without lymph node metastasis, and the EMT and stemness of BCCs with lymph node metastasis were more active than those of BCCs without lymph node metastasis.

### CAV-1-downregulated fibroblasts enhance the migration and invasion ability of breast cancer cells

**Figure 2:**
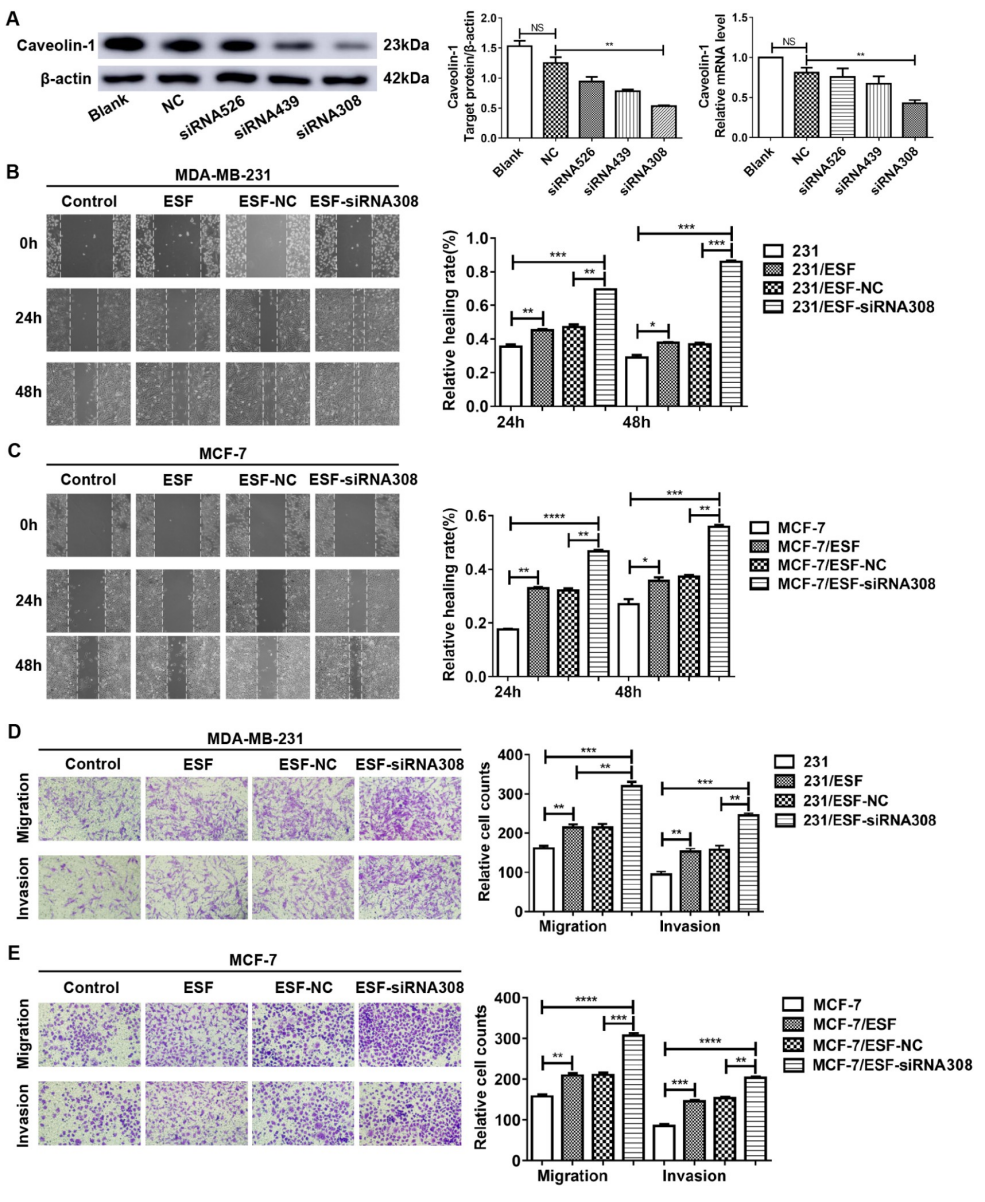
Figure 2 CAV-1 deficiency in fibroblasts promotes the migration and invasion ability of BCCs (A) Western blot analysis was performed to detect the expression level of CAV-1 in fibroblasts after transfection with different siRNAs. (B,C) Cell scratch test was conducted to detect the migration ability of MDA-MB-231 and MCF-7 cells. (D,E) Transwell assays were conducted to measure the migration and invasion abilities of MDA-MB-231 and MCF-7 cells. Data are shown as the mean±SD, n=3. * P<0.05, ** P<0.01, and *** P<0.001, compared with the corresponding controls.

To evaluate the effects of CAV-1-downregulated fibroblasts on the metastasis of breast cancer cells, three kinds of siRNA (siRNA536, siRNA439, and siRNA308) were designed and transfected into fibroblasts to knockdown CAV-1 expression, The results showed that siRNA308 was the most effective one ( Figure 2A). We performed the cell scratch test and Transwell assay to detect BCC migration and invasion ability. As shown in the cell scratch test, coculture with normal ESFs enhanced the migration ability of MDA-MB-231 ( Figure 2B) and MCF-7 cells ( Figure 2C) compared with cancer cells cultured alone. CAV-1-downregulated ESF cells further enhanced the migration ability of BCCs. In the Transwell assay, coculture with normal ESFs enhanced the migration and invasion abilities of MDA-MB-231 ( Figure 2D) and MCF-7 cells ( Figure 2E) compared with cancer cells cultured alone. Moreover, this effect could be further promoted by co-culture with CAV-1-downregulated ESFs. Additionally, the migration and invasion abilities of MDA-MB-231 cells were significantly higher than those of MCF-7 cells.

### CAV-1-downregulated fibroblasts promote EMT and stemness of breast cancer cells

**Figure 3:**
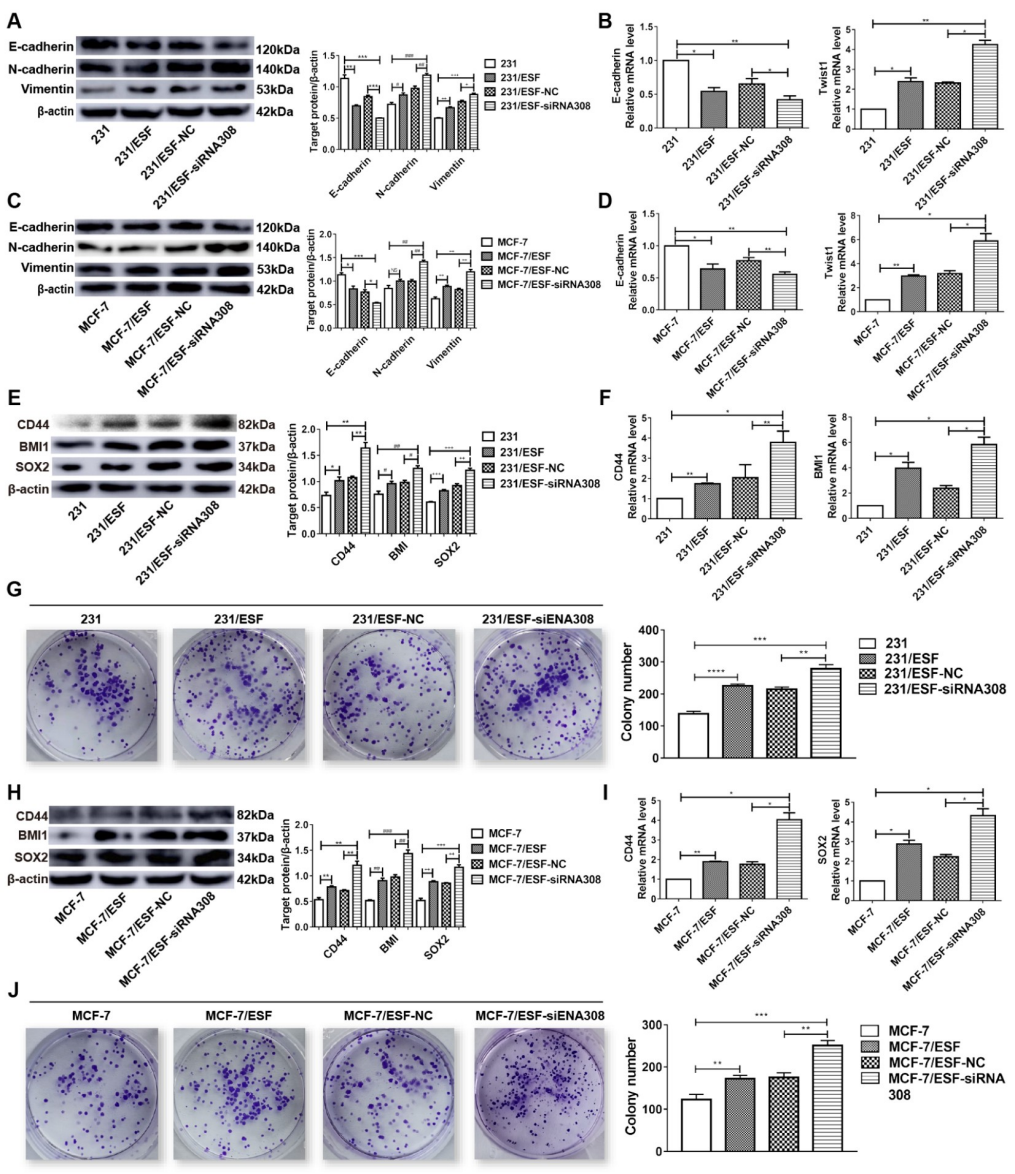
Figure 3 CAV-1 deficiency in fibroblasts promotes EMT and stemness of BCCs Western blot analysis and RT-qPCR were used to measure the expression of EMT and stemness markers in MDA-MB-231 and MCF-7 cells. Colony formation assay was performed to confirm the stemness of BCCs, and the plating efficiency was determined from a number of stained colonies. EMT (A–D) and stemness (E–J) were enhanced in cocultured BCCs, and CAV-1 deficiency in ESFs could enhance these differences. Data are shown as the mean±SD, n=3. * P<0.05, ** P<0.01, *** P<0.001, **** P<0.0001, # P<0.05, ## P<0.01, ### P<0.001, + P<0.05, ++ P<0.01, and +++ P<0.001, compared with the corresponding controls.

As shown in the aforementioned results, CAV-1 downregulation in ESFs promoted the migration and invasion capacity of BCCs. To explore whether EMT and stemness participate in this process, we cocultured MDA-MB-231 and MCF-7 cells with normal ESFs or siRNA308-transfected ESFs. RT-qPCR and western blot analysis were used to measure EMT and stemness markers in BCCs, including E-cadherin, N-cadherin, Vimentin, CD44, BMI1, and SOX2. Colony formation assay was performed to detect the stemness of BCCs, and the colony formation rate was calculated. The results from the western blot analysis ( Figure 3A,C) and RT-qPCR ( Figure 3B,D) showed that coculture with ESFs enhanced the EMT of MDA-MB-231 and MCF-7 cells. Moreover, compared to coculture with normal ESFs, coculture with CAV-1-deficient ESFs further promoted cancer cell EMT. Additionally, western blot analysis ( Figure 3E,H), RT-qPCR ( Figure 3F,I), and colony formation assays ( Figure 3G,J **)** also revealed that coculture with ESFs enhanced BCC stemness and that CAV-1 deficiency in ESFs further enhanced this difference. All of these results suggested that CAV-1 downregulation in ESFs might be an intermediate step of EMT and stemness in BCCs.

### CAV-1 deficiency can induce ESF to secrete TGF-β1 to activate the TGF-β/Smad signaling pathway in BCCs

**Figure 4:**
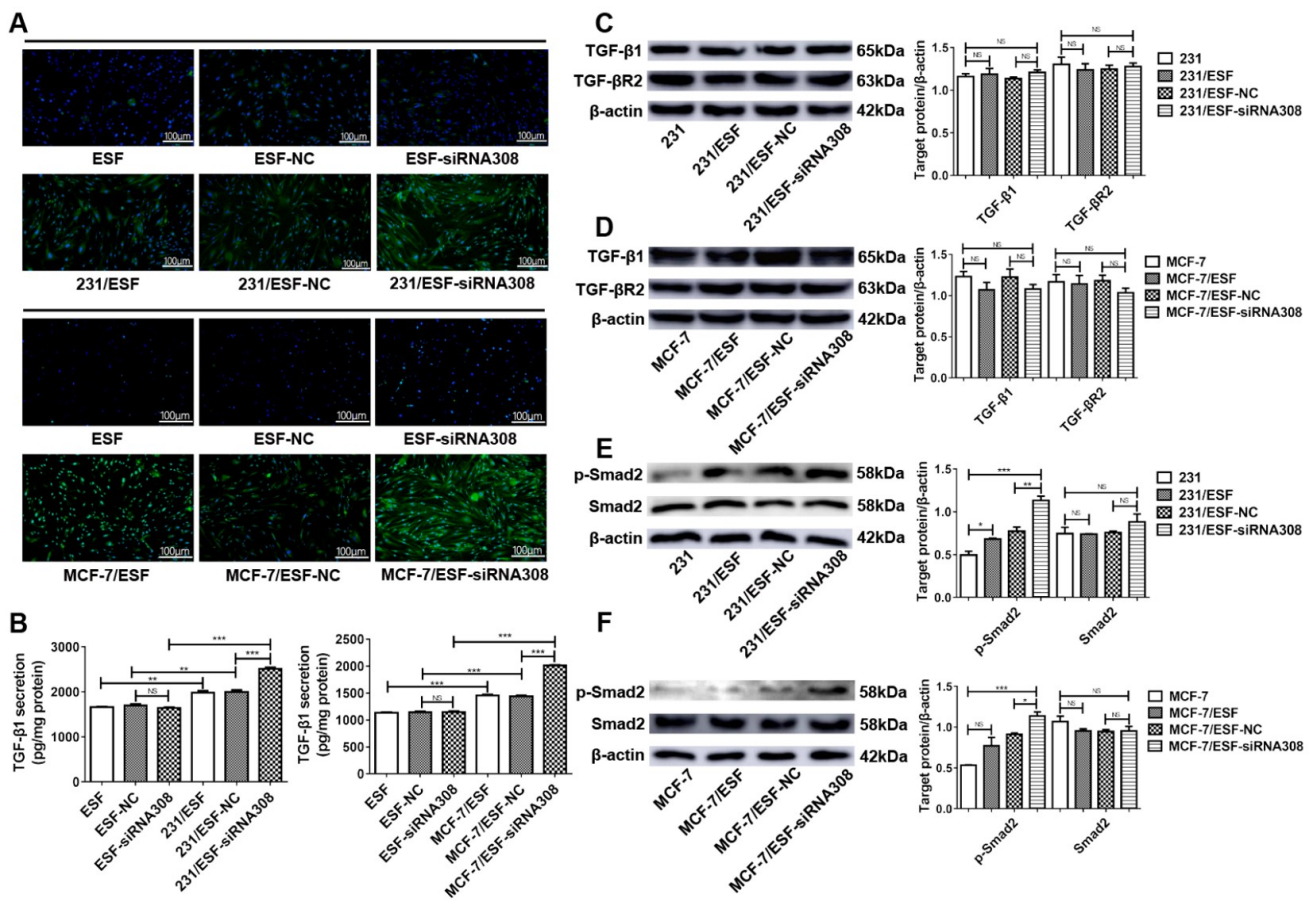
Figure 4 CAV-1 deficiency in fibroblasts induces TGF-β1 secretion and activates the TGF-β/Smad signaling pathway in BCCs Immunofluorescence staining (A) and ELISA (B) were performed to detect TGF-β1 expression and secretion levels of fibroblasts in different groups. Western blot analysis (C–F) was used to measure the TGF-β1, TGF-βR2, p-Smad, and Smad expressions in BCCs in the single or cocultured groups. Data are shown as the mean±SD, n=3. * P<0.05, ** P<0.01, *** P<0.001, # P<0.05, ## P<0.01, ### P<0.001, + P<0.05, ++ P<0.01, and +++ P<0.001, compared with the corresponding controls.

To investigate the exact mechanism by which ESF promotes BCC stemness and EMT, we performed immunofluorescence staining and ELISA to measure TGF-β1 expression in ESFs and secretion levels in the cocultured medium. The immunofluorescence staining results showed that TGF-β1 expression was upregulated in the cocultured ESFs and that the expression level was significantly boosted when CAV-1 was downregulated in ESFs ( Figure 4A). According to the ELISA results, TGF-β1 secretion was enhanced when ESFs were cocultured with BCCs, and CAV-1 deficiency in ESFs further enhanced this difference ( Figure 4B). However, the TGF-β1 secretion level remained almost unchanged between these noncocultured ESFs transfected with siRNA308.

In addition, western blot analysis was performed to measure the TGF-β1, TGF-βR2 ( Figure 4C,D), Smad, and phosphorylated (p)-Smad2 ( Figure 4E,F) expression levels in BCCs. The results showed that TGF-β1, TGF-βR2, and Smad expression were not significantly different between cells cocultured with normal or CAV-1-deficient ESFs. p-Smad expression in BCCs was upregulated when cocultured with ESFs, and CAV-1 deficiency in ESFs further boosted it. These results indicate that coculture with BCCs can improve TGF-β1 expression and secretion of ESF, and CAV-1 deficiency in ESF can promote it further. Meanwhile, TGF-β1 secreted from ESF can activate the TGF-β/Smad pathway in BCCs.

### SB431542 inhibits the EMT and stemness of BCCs in a CAV-1-deficient microenvironment by inhibiting the TGF-β1/Smad signaling pathway

**Figure 5:**
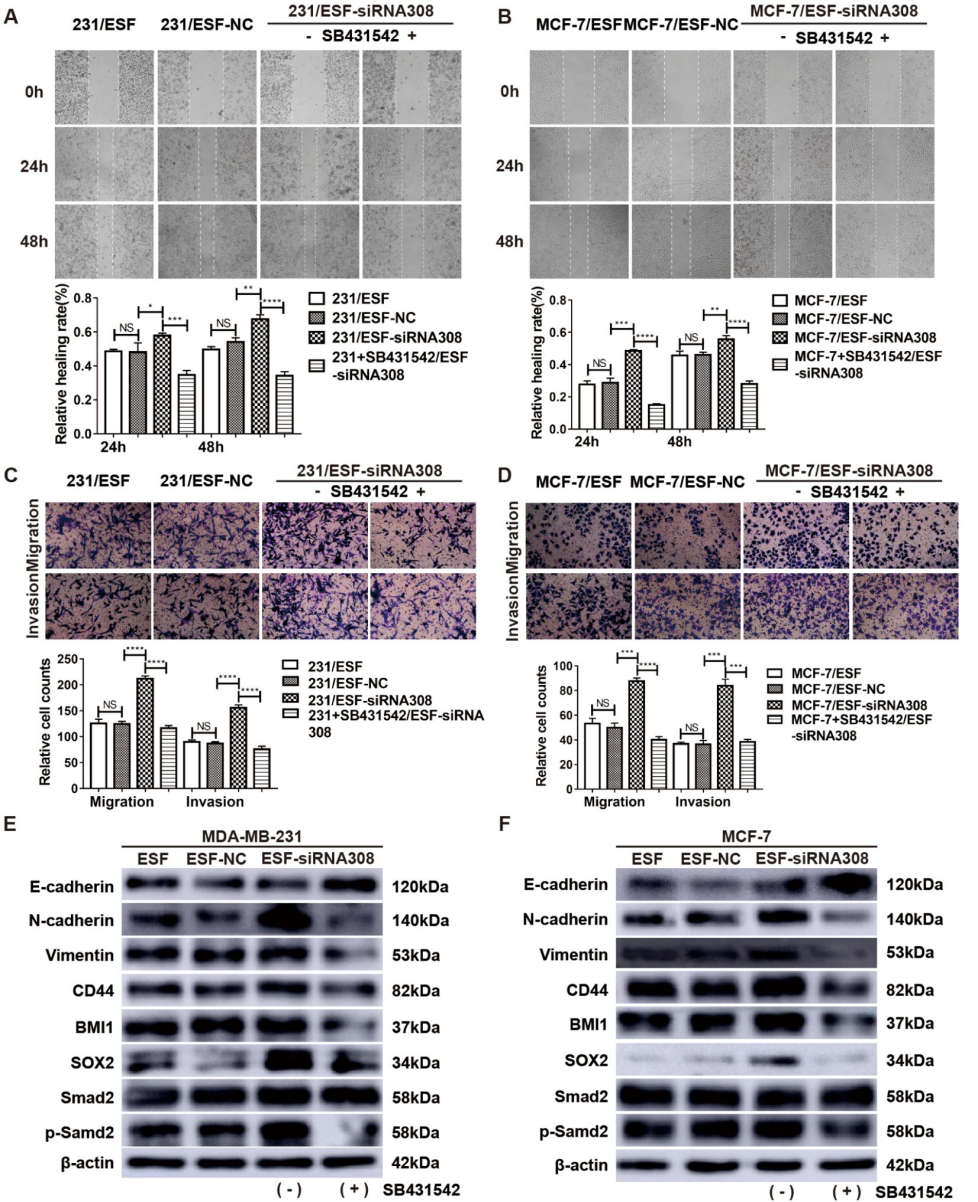
Figure 5 SB431542 inhibits EMT and stemness of BCCs in the CAV-1-deficient microenvironment by inhibiting the TGF-β1/Smad signaling pathway Cell scratch test (A,B) and Transwell assay (C,D) were performed to measure the migration and invasion ability of the cocultured BCCs with or without SB431542 treatment. Western blot analysis (E,F) was used to detect EMT- and stemness-related protein and signaling protein (Smad and p-Smad) expression levels in the cocultured BCCs. Data are shown as the mean±SD, n=3. * P<0.05, ** P<0.01, *** P<0.001, **** P<0.0001, # P<0.05, ## P<0.01, ### P<0.001, compared with the corresponding controls. NS, no significance.

SB431542 is a newly discovered molecular inhibitor of the TGF-β/Smad signaling pathway, which can conspicuously inhibit the phosphorylation and nuclear translocation of Smad, thereby impeding TGF-β-mediated transcription [31]. Therefore, cell scratch test ( Figure 5A,B) and Transwell assay ( Figure 5C,D) were used to verify whether the migration and invasion ability of BCCs in the CAV-1-deficient microenvironment could be reversed after treatment with SB431542. Western blot analysis results showed that the expression levels of proteins involved in TGF-β signaling (Smad2 and p-Smad2) and the biomarkers of EMT and stemness of the ESF-cocultured MDA-MB-231 and MCF-7 cells were also restrained after treatment with 1.5 mM and 1.0 mM SB431542 , respectively ( Figure 5E,F). These results indicated that the migration, invasion ability, and stemness of BCCs cocultured with CAV-1-downregulated ESFs were inhibited after SB431542 was used to block the TGF-β/Smad signaling in BCCs.

## Discussion

Breast cancer is the most common cancer among women, and its recurrent distant metastasis is one of the most important causes of deterioration and death in breast cancer patients. Cancer cell migration and invasion are key steps in the aforementioned distant metastasis, which is functionally dependent on the EMT and stemness of CSCs, among other factors.

Recently, many researchers suggested that CAFs can regulate different physiological functions of tumor cells, and according to the analyzed data of GEPIA, the CAV-1 expression level is significantly downregulated in breast cancer tissues compared with that in normal tissues. Some previous investigations indicated that CAV-1 expression in CAFs has a positive relationship with a better prognosis of breast cancer patients [ 32, 33] . In addition, the IHC and WB results of collected breast cancer tissues indicated that tumor-stromal CAV-1 expression is reduced, while the EMT and stemness of BCCs were activated, in cancer tissues with lymph node metastasis, but the exact mechanism remains ambiguous. Therefore, we cocultured ESFs with MDA-MB-231 or MCF-7 cells to simulate the microenvironment of breast cancer tissue, aiming to reveal the specific changes in ESFs.

We established siRNA308 to knockdown CAV-1 expression in ESFs to simulate the CAV-1-deficient microenvironment. After co-culture with CAV-1-deficient fibroblasts, the invasion and migration capacity of BCCs was increased. Meanwhile, the tumor sphere formation ability of BCCs was enhanced after transfection with siRNA308 to block CAV-1 expression in the co-cultured fibroblasts compared with that in the BCCs in the single culture and ESF-NC groups. These results showed that the EMT and stemness of BCCs were activated. Previous studies reported that CAFs can not only secrete leptin to maintain the self-renewal and invasion of BCCs but also exude interleukin-6 (IL-6) and interleukin-8 (IL-8) to promote BCC proliferation by activating the nuclear factor kappa-B (NF-κB)/p65 signaling pathway [ 34– 36] . Moreover, CAFs can secrete exosomes that act on BCCs and colorectal cancer cells by transferring microRNAs to regulate the stemness and EMT of cancer cells [ 37– 39] and promote tumor growth, invasion, and metastasis [40]. In addition to directly affecting the EMT and stemness of tumor cells, CAFs participate in regulating tumor cells by secreting components of the extracellular matrix (ECM) [ 41, 42] . For example, in triple-negative breast cancer, CAFs can correspond to the variation of Hedgehog in tumor cells through secreting fibroblast growth factor 5, subsequently reconstructing the ECM [43]. On the other hand, the tumor microenvironment plays an essential role in cancer cell migration and invasion, and cancer cells can also induce CAF differentiation to remodel the ECM by secreting TGF-β and extracellular vesicles containing miRNA192/215 to inhibit CAV1 expression and activate the TGF-β/Smad pathway in fibroblasts [44]. All of these investigations imply that CAFs and the communication between them and BCCs in the tumor microenvironment play a crucial role in the progression of breast cancer.

Previous studies have demonstrated that the TGF-β signaling pathway also plays a positive role in the self-renewal, differentiation, and proliferation of tumor stem cells, including breast cancer [ 45, 46] , hepatocellular carcinoma [47], gastric cancer [48], and prostate cancer [49]. In the late stage of tumorigenesis, TGF-β/Smad signaling induces tumor metastasis and invasion by regulating some transcription factors (TFs) related to EMT [ 50, 51] . According to the above findings, we demonstrated that coculture with BCCs enhanced TGF-β1 secretion of ESF, which activates the TGF-β/Smad pathway in BCCs, and CAV-1 deficiency accelerated this process. However, the level of TGF-β1 secreted by ESFs (without BCC coculture) did not change significantly, while it remained after the downregulation of CAV-1. These results indicated that the secretion of TGF-β1 by ESF mainly relied on the tumor microenvironment. Interestingly, some feedback regulation mechanisms might be involved in this process, such as exporter-recipient communication by targeting TGF-β ligands, receptors, or other transcriptions in the cell plasma and microenvironment [52]. More *in vitro* and *in vivo* experiments are required to illustrate the underlying mechanisms.

Due to the close relationship among CAFs, cancer progression, and the TGF-β signaling pathway, we used SB431542 to block the TGF-β signaling pathway in BCCs, which verified that the migration and invasion abilities of BCCs in the CAV-1-deficient microenvironment were reversed after treament with SB431542. As expected, the upregulation of EMT marker expressions was reversed, and the expressions of CSC-related markers were also decreased. These results thus confirm that the deficiency of CAV-1 in fibroblasts promotes breast cancer progression from the opposite direction.

**Figure 6:**
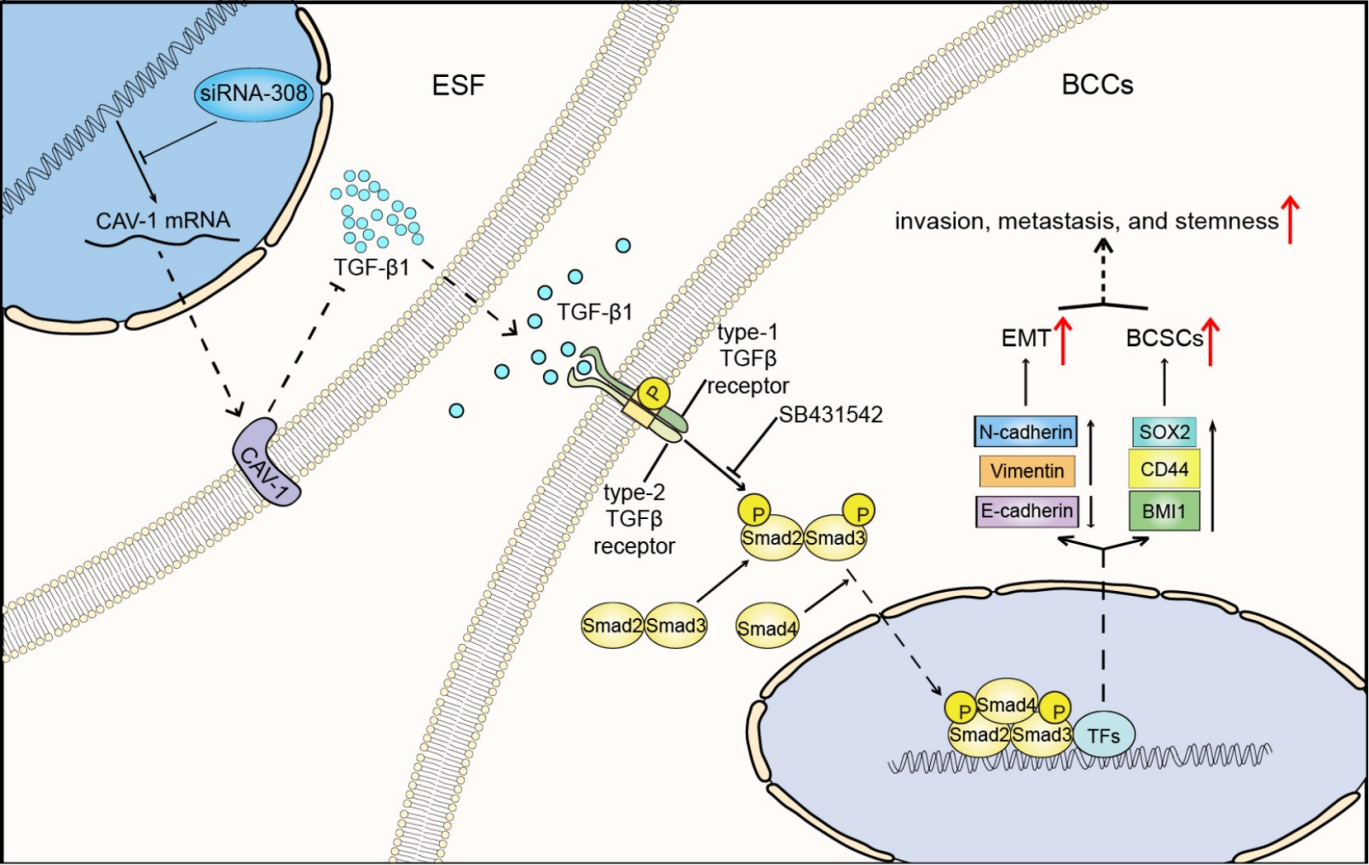
Figure 6 CAV-1 deficiency in fibroblasts promotes the invasion, migration, and stemness of BCCs by activating the TGF-β1/Smad signaling pathway Interference with siRNA308 downregulates CAV-1 expression in ESFs and promotes TGF-β1 secretion. TGF-β1 subsequently activates the TGF-β1/Smad signaling pathway in BCCs and regulates associated genes to promote EMT and stemness.

To summarize, our study demonstrates how CAV-1 downregulation affects the invasiveness and metastasis of breast cancer cells ( Figure 6). Through interference with siRNA308, downregulation of CAV-1 in fibroblasts promotes TGF-β1 secretion and activates TGF-βR2 in BCCs, which enhances the phosphorylation of Smad2 and its combination with Smad4 after translocation to the nucleus. Finally, its downstream signaling promotes the EMT and stemness of BCCs by regulating associated genes and other TFs, resulting in the enhanced invasiveness and metastasis of BCCs. However, further research is needed to answer the following questions: Is CAV-1 downregulation in CAFs correlated with decreased gene expression or increased degradation? How do BCCs promote the secretion of TGF-β1 by CAFs, and does the microenvironment play any role in this process? Addressing these key issues would further corroborate the mechanism of CAV-1 in breast cancer progression.

## Supplementary Data

Supplementary data is available at *Acta Biochimica et Biophysica Sinica* online.
