# Corrigendum to “Caveolin-1-deficient fibroblasts promote migration, invasion, and stemness by activating the TGF-β/Smad signaling pathway in breast cancer cells”: Corrigendum

**DOI:** 10.3724/abbs.2025233

**Published:** 2025-12-25

**Authors:** Qingyun Huang, Longyuan Wu, Yi Wang, Xinyu Kong, Xinhua Xiao, Qiyuan Huang, Miao Li, Yujia Zhai, Fuxiu Shi, Ruichen Zhao, Junpei Zhong, Lixia Xiong

*Acta Biochim Biophys Sin* 54: 1587–1598. doi: 10.3724/abbs.2022150 . 

In the course of a detailed, post-publication review of the article, the authors identified inaccuracies in the preparation of several figures that require rectification. The authors are providing this corrigendum to ensure the complete accuracy of the scientific record.

The specific errors are as follows:

**1. Figure 2D** was inadvertently mislabeled during preparation. The authors have now replaced it with the correct version. 

**2. Figure 4A** contained an incorrect image due to a processing error. The updated figure accurately represents the experimental results. 

**3. Figure 5A** was mistakenly replaced during the final compilation. The authors have provided the correct image for this panel. 

The authors have verified that these errors are strictly confined to the figure presentation and do not impact the underlying data, statistical analysis, or the main conclusions drawn in the manuscript. The authors deeply apologize for this oversight and any resulting inconvenience to the readers.

Corrected Figure 2D

**Figure:**
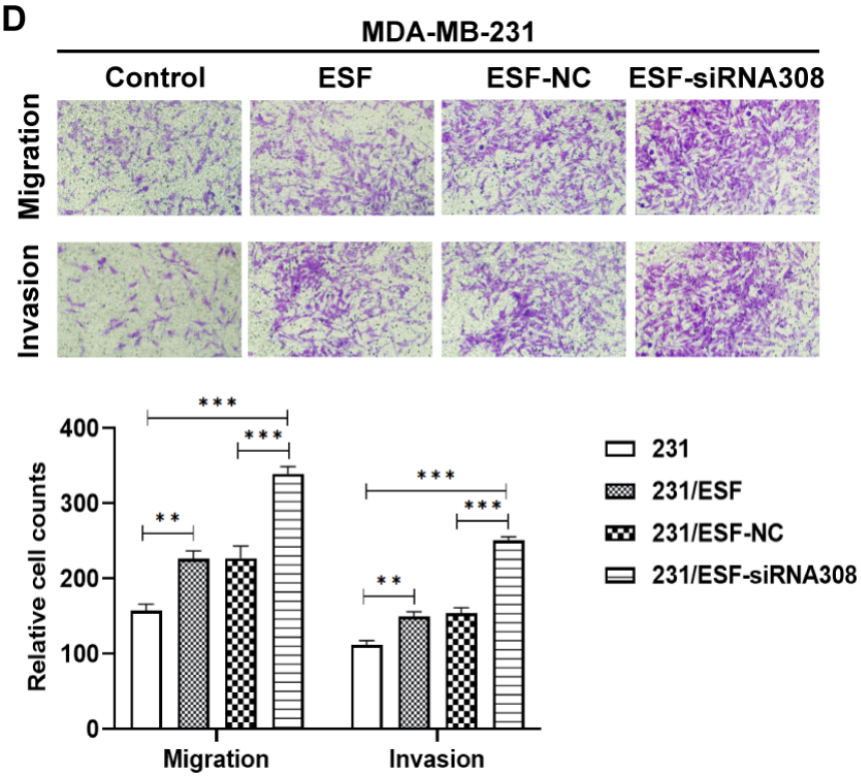
Figure 2 CAV-1 deficiency in fibroblasts promotes the migration and invasion ability of BCCs Western blot (A) was performed to detect the expression level of CAV-1 in fibroblasts, after transfecting different siRNA. Cell scratch test was conducted to detect the migration ability of MDA-MB-231 (B) and MCF-7 (C) cells. Transwell assay was conducted to measure the migration and invasion ability of MDA-MB-231 (D) and MCF-7 (E) cells. Data are shown as the mean ± SD, n = 3. Values were significantly different compared with the corresponding control value at *P < 0.05, **P < 0.01, ***P < 0.001.

Corrected Figure 4A

**Figure:**
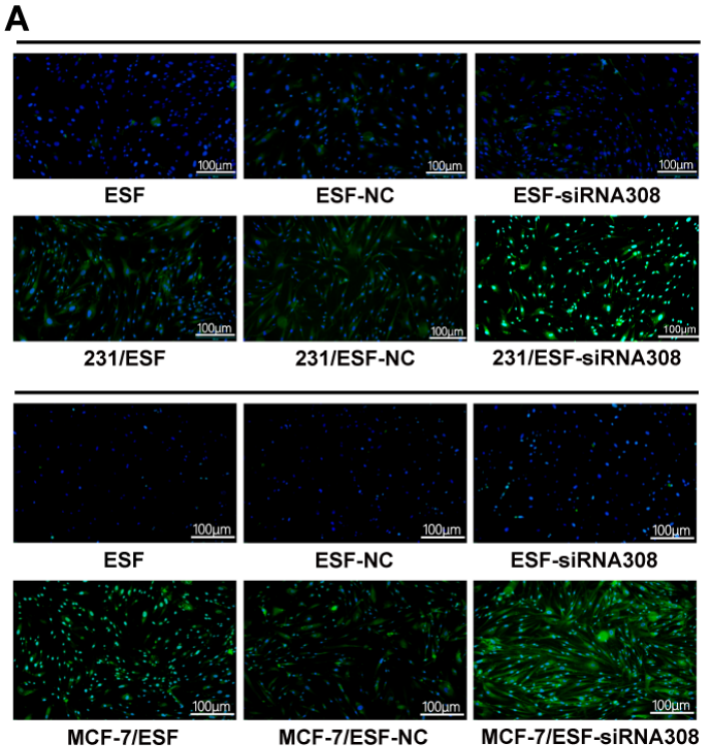
Figure 4 CAV-1 deficiency in fibroblast induces TGF-β1 secretion and activate the TGF-β/Smad Signaling Pathway in BCCs Immunofluorescence staining (A) and ELISA (B) was performed to detect the TGF-β1 expression and secretion level of fibroblast in different groups. Western blot (C-F) was used to measure the TGF-β1, TGF-βR2, p-Smad, and Smad expressions of BCCs in single or co-cultured groups. Data are shown as the mean ± SD, n = 3. Values were significantly different compared with the corresponding control value at *P < 0.05, **P < 0.01, ***P < 0.001; # P < 0.05, ##P < 0.01, ### P < 0.001; +P < 0.05, ++ P < 0.01, +++P < 0.001.

Corrected Figure 5A

**Figure:**
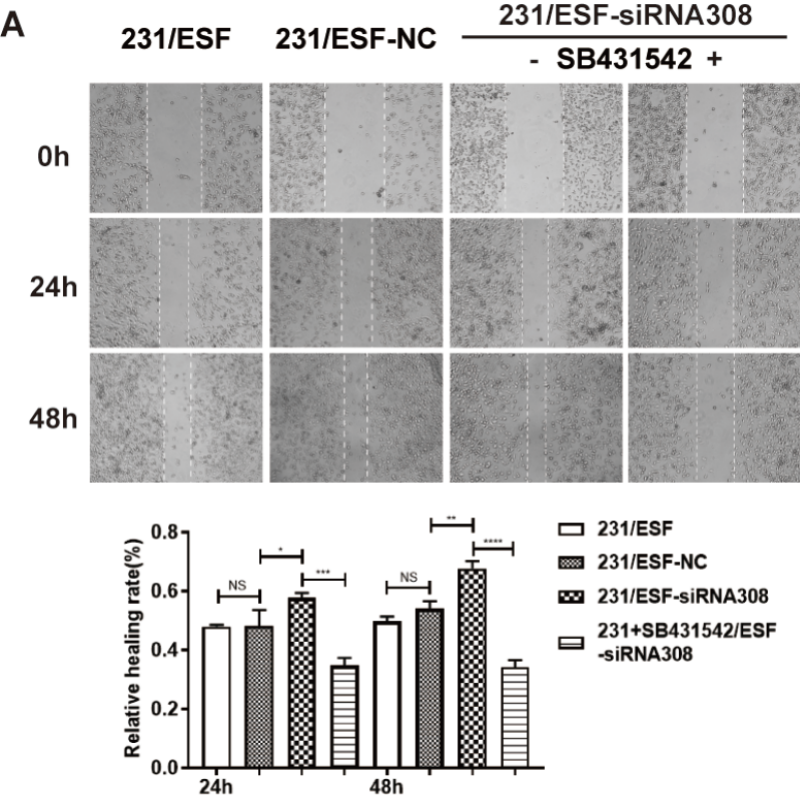
Figure 5 SB431542 inhibits EMT and stemness of BCCs in the CAV-1-deficient microenvironment via inhibiting the TGF-β1/Smad signaling pathway Cell scratch test (A,B) and Transwell assay (C,D) were established to measure the migration and invasion ability of the cocultured BCCs with or without administrating SB431542. Western blot (E,F) was used to detect the EMT and stemness-related proteins, and signaling proteins (Smad and p-Smad) expression level in the cocultured BCCs. Data are shown as the mean ± SD, n = 3. Values were significantly different compared with the corresponding control value at *P < 0.05, **P < 0.01, ***P < 0.001, ****P < 0.0001; #P < 0.05, ##P < 0.01, ###P < 0.001.

